# Live Attenuated African Swine Fever Viruses as Ideal Tools to Dissect the Mechanisms Involved in Cross-Protection

**DOI:** 10.3390/v12121474

**Published:** 2020-12-21

**Authors:** Elisabeth Lopez, Juanita van Heerden, Laia Bosch-Camós, Francesc Accensi, Maria Jesus Navas, Paula López-Monteagudo, Jordi Argilaguet, Carmina Gallardo, Sonia Pina-Pedrero, Maria Luisa Salas, Jeremy Salt, Fernando Rodriguez

**Affiliations:** 1 IRTA, Centre de Recerca en Sanitat Animal (IRTA-CReSA), Campus de la Universitat Autonoma de Barcelona, 08193 Bellaterra, Spain; elisabeth.lopezf@gmail.com (E.L.); laia.bosch@irta.cat (L.B.-C.); francesc.accensi@irta.cat (F.A.); mariajesus.navas@irta.cat (M.J.N.); paulalopezmonteagudo@gmail.com (P.L.-M.); jordi.argilaguet@irta.cat (J.A.); sonia.pina@irta.cat (S.P.-P.); 2 Agricultural Research Council-Onderstepoort Veterinary Research, Pretoria 0110, South Africa; VanHeerdenJ@arc.agric.za; 3 Departament de Sanitat i d’Anatomia Animals, Facultat de Veterinària, UAB, 08193 Bellaterra, Spain; 4 Centro de Investigación en Sanidad Animal (CISA-INIA), 28130 Madrid, Spain; gallardo@inia.es; 5 Centro de Biología Molecular Severo Ochoa, Consejo Superior de Investigaciones Científicas and Universidad Autònoma de Madrid, 28049 Madrid, Spain; mlsalas@cbm.csic.es; 6 GALVmed, Doherty Building, Pentlands Science Park, Bush Loan, Penicuik Edinburgh EH26 0PZ, UK; Jeremy.Salt@galvmed.org

**Keywords:** African swine fever virus, live attenuate vaccine, cross-protection

## Abstract

African swine fever (ASF) has become the major threat for the global swine industry. Furthermore, the epidemiological situation of African swine fever virus (ASFV) in some endemic regions of Sub-Saharan Africa is worse than ever, with multiple virus strains and genotypes currently circulating in a given area. Despite the recent advances on ASF vaccine development, there are no commercial vaccines yet, and most of the promising vaccine prototypes available today have been specifically designed to fight the genotype II strains currently circulating in Europe, Asia, and Oceania. Previous results from our laboratory have demonstrated the ability of BA71∆CD2, a recombinant LAV lacking CD2v, to confer protection against homologous (BA71) and heterologous genotype I (E75) and genotype II (Georgia2007/01) ASFV strains, both belonging to same clade (clade C). Here, we extend these results using BA71∆CD2 as a tool trying to understand ASFV cross-protection, using phylogenetically distant ASFV strains. We first observed that five out of six (83.3%) of the pigs immunized once with 10^6^ PFU of BA71∆CD2 survived the tick-bite challenge using *Ornithodoros* sp. soft ticks naturally infected with RSA/11/2017 strain (genotype XIX, clade D). Second, only two out of six (33.3%) survived the challenge with Ken06.Bus (genotype IX, clade A), which is phylogenetically more distant to BA71∆CD2 than the RSA/11/2017 strain. On the other hand, homologous prime-boosting with BA71∆CD2 only improved the survival rate to 50% after Ken06.Bus challenge, all suffering mild ASF-compatible clinical signs, while 100% of the pigs immunized with BA71∆CD2 and boosted with the parental BA71 virulent strain survived the lethal challenge with Ken06.Bus, without almost no clinical signs of the disease. Our results confirm that cross-protection is a multifactorial phenomenon that not only depends on sequence similarity. We believe that understanding this complex phenomenon will be useful for designing future vaccines for ASF-endemic areas.

## 1. Introduction

Long before the importation of domestic pigs (*Sus scrofa*) to Africa, African swine fever virus (ASFV) circulated between their natural reservoirs, *Ornithodoros moubata* complex ticks and African wild pigs, mostly warthogs (*Phacochoerus africanus*), without causing any apparent disease [1]. African swine fever (ASF) was described for the first time in Kenya in 1921 as a fatal hemorrhagic disease, affecting exclusively domestic pigs. Since then, it has contributed to the underdevelopment of ASF-endemic areas in Sub-Saharan Africa [2,3]. ASFV gained notoriety with its consecutive exportations to Portugal in 1957 and 1960, remaining endemic in the Iberian Peninsula for almost 40 years, provoking a tremendous economic impact to the affected areas. During this endemic period, ASFV affected different countries of South America and Europe. However, the virus was efficiently controlled by means of stamping out policies, with the exception of Sardinia, where ASFV remains endemic since 1978. ASFV is included in the list of diseases of obliged declaration to the World Organization for Animal Health (OIE). In 2007, just ten years after declaring continental Europe an ASF-free zone [4], the virus was reintroduced into the continent, this time through the port of Poti in Georgia [5]. From this port, ASFV has expanded east and west, being present today in 28 countries from Europe, Asia, and Oceania, according to the data reported to the OIE [6]. In addition, ASF outbreaks are constantly being declared in different countries of sub-Saharan Africa. Consequently, ASF has become the number one threat for the swine industry, affecting the global commerce equilibrium [7].

A lack of safe and efficient vaccines complicates the control of ASF, preventing any immediate eradication program. Today, several vaccine candidates are available, all based on live attenuated viruses (LAVs), with real potential to become the first commercial vaccine in the short–medium term [8,9,10]. Arbitration of regulatory agencies will guarantee that only the safest LAVs reach the field. Most of these vaccines have been designed to specifically target the genotype II ASFV strains currently circulating in Europe and Asia, where, so far, the virus seems to circulate without significant genetic changes [11]. A similar picture has been described for the genotype I virus circulating in Sardinia since 1978 until today, most probably reflecting the fact that a single virus incursion has occurred during the entire endemic period [12]. Agreeing with this hypothesis, the introduction of ASFV in the Iberian Peninsula in at least two different occasions could contribute to explain the multiple non cross-protective or heterologous ASFV strains described in this area during the endemic period [13], therefore complicating vaccine designing. ASFV genome variability reaches its maximal expression in Africa, where 24 different genotypes have been described so far, divided into four geographical clades [14]. Taking into account the lack of natural cross-protection that exists between circulating viruses in Africa [15], designing future vaccines for the area will be specially challenging. We believe that deeply understanding the mechanisms governing cross-protection deserves further attention. Here, we propose BA71∆CD2, a recombinant LAV lacking CD2v [16], as a tool to study ASFV cross-protection. Preliminary work from our laboratory demonstrated the protective potential of BA71∆CD2 against the experimental challenge with the homologous, BA71, and the heterologous, E75, virulent strains of ASFV (both genotype I ASFV strains), and against the heterologous strain Georgia2007/1 (genotype II). All three of the latter strains belong to the same genomic clade (clade C). In this study, we are using phylogenetically distant ASF strains from clades D and A. Our preliminary results confirm cross-protection as a multifactorial phenomenon that depends not only on sequence similarity but also on complex immunological reactions, which most probably involve innate and adaptive (both humoral and cellular) responses. Our results confirm live attenuated ASFV as tools to dissect the mechanisms involved in cross-protection, which is crucial for the future designing of vaccines for endemic areas with complex ASFV epidemiological situations.

## 2. Materials and Methods

### 2.1. Viruses and Cells

BA71∆CD2, is a recombinant live attenuated virus, lacking CD2v, the ASFV hemagglutinin that is capable of protecting pigs against the homologous BA71 (genotype I, clade C) virus, the heterologous E75 (genotype I, clade C) virus, and the Georgia2007/1 (genotype II, clade C) virus, which today are globally circulating [16]. Four different ASFV virulent strains were used: BA71, isolated in Spain in 1971, Ken06.Bus (genotype IX, clade A), isolated in Kenya in 2006 [17], RSA/11/2017 (genotype XIX, clade D), isolated form *Ornithodoros* sp ticks captured in South Africa, and Georgia2007/1, isolated in Georgia in 2007. Except for Georgia2007/1, which is exclusively used for in vitro experiments, all ASFV strains were used in vivo.

Field isolates were grown in swine alveolar macrophages, and BA71∆CD2 was grown in COS-1 cells [16].

### 2.2. Animal Welfare

Large white pigs ranging from 15 to 30 kg were used in all the experiments. Animal experiments were conducted strictly according to animal welfare ethics and protocols were approved by the Ethics Committees from either, the Agricultural Research Council (ARC), Onderstepoort Veterinary Research, (OVR), or the Institut de Recerca i Tecnologia Agroalimentària (IRTA) from Catalonia.

### 2.3. Experimental Approach

A total of three in vivo experiments were performed in two different locations.

For the first experiment, located in Onderstepoort Veterinary Research facilities, a group of six pigs were intramuscularly (IM) immunized with 10^6^ Plaque Forming Units (PFU) of BA71∆CD2, and two additional pigs remained un-immunized as controls. Sixteen days after immunization, pigs were challenged with field-harvested ticks naturally infected with the RSA/11/2017 virus. Briefly, *Ornithodoros* sp soft ticks were collected in a warthog burrow from a private game farm in the Dinokeng area, north of Pretoria, Gauteng Province (South Africa), previously defined as an ASF-free zone according to a modified manual collection method [18]. PCR sequencing allowed identifying a pure population of ASFV [19], which according to a neighbor joining phylogenetic tree constructed in Mega 5.1 [20], perfectly matched the genotype XIX. Ninety-six ticks ranging between N2 and adult were randomly divided into eight groups of 12 ticks each to perform the infection experiment without knowing the ASFV titer harbored by the ticks. Ticks were allowed to feed on the hip of the pigs until the ticks dropped off or up to a maximum feeding time of 60 min.

The second and third in vivo experiments were host in the Biosafety level 3 plus facilities (BSLA3+) at IRTA-CReSA. Initially, two groups of six pigs were IM immunized with 3.3 × 10^4^ or 10^6^ PFU of BA71∆CD2, respectively, and three additional un-immunized pigs served as controls. Three weeks after the immunization, pigs were IM challenged with a lethal dose of 10^2^ HAU (Hemagglutination Units) of Ken06.Bus strain.

For the last experiment, a group of four pigs was IM immunized twice, three weeks apart, with 3.3 × 10^4^ PFU of BA71∆CD2. A second group of four pigs was IM immunized first with 3.3 × 10^4^ PFU of BA71∆CD2 and 21 days later, it was boosted with an IM lethal dose of 10^3^ HAU of the BA71 virulent strain, following similar heterologous prime-boosting experiments performed before using other LAVs [13,21]. Three extra pigs remained un-immunized as controls. Then, all animals were challenge with a lethal dose of 10^2^ HAU of Ken06.Bus, 42 days after first immunization.

All pigs were bled, and nasal swaps and rectal temperatures were taken from the day of RSA/11/2017 or Ken06.Bus challenge. Animals were observed daily according to the welfare schedule to monitor their health status and or record the clinical signs after the infection with ASFV [22]. Post-mortem examinations were carried out to confirm or discard the presence of ASFV-compatible pathological lesions.

### 2.4. Analytical Readouts

Experimental immunization, clinical observations, immunological assays, and virus titration methods were performed as previously described [16], with few exceptions.

#### 2.4.1. ASFV Quantification by Real-Time PCR

Sera and nasal swabs collected from the day of RSA/11/2017 or Ken06.Bus challenge were used to quantify the viral DNA by real-time PCR (qPCR). Briefly, the viral genomic DNA was obtained from 200 µL of sera or swabs-PBS suspension using the Nucleospin Blood kit (Macherey-Nagel) and then used as template to amplify an 85 bp-long fragment from the ASFV serine protein kinase gene (R298L). PCR amplifications were performed in duplicates using corresponding standards for absolute quantification. The results were expressed as log10 genome equivalent copies (GEC) per ml of sera or nasal swab. The detection limit of the technique was set at 10^3^ GEC/mL.

#### 2.4.2. Antibody Detection by ELISA

In general, ASFV-specific antibodies in pig sera were detected by the OIE-approved ELISA assay based on soluble extracts from ASFV-infected cells. The presence of positive sera was detected using a peroxidase-conjugated anti-pig immunoglobulin G (IgG) at 1:20,000 dilution (Sigma-Aldrich) or Anti-pig immunoglobulin A (IgA) conjugated with peroxidase 1:10,000 as secondary antibodies and soluble 3,3′,5,5′-tetramethylbenzidine (TMB) as specific peroxidase substrate (Sigma-Aldrich). Reactions were stopped with 1 N H_2_SO_4_ (Sigma-Aldrich), and the ELISA plates were read at 450 nm wavelength (λ450).

To detect ASFV-specific IgG1 and IgG2 pig antibodies, the ELISA was performed using mouse anti-pig monoclonals (Sigma-Aldrich) diluted at 1/5000, followed by goat anti-mouse immunoglobulins conjugated with peroxidase 1/10,000. ASFV-specific IgG1/IgG2 ratios were calculated with optical density OD values at 450 nm, once normalized with respect to the OD value of positive control wells. 

During the tick-bite challenge experiment performed in South Africa, antibodies against ASFV protein VP72/B646L were measured using a commercial competitive ELISA (INGEZIM PPA Compac, Ingenasa, Madrid, Spain).

#### 2.4.3. T Cell-Specific Immune Response

The frequency of ASFV-specific interferon gamma secreting cells (IFNγ-SC) in PBMCs, was analyzed by an Enzyme-linked Immunosorbent Spot (ELISPOT) assay using commercial monoclonal antibody tandems (swine IFNγ; Cytoset). Briefly, peripheral blood mononuclear cells (PBMCs) were isolated from whole blood by density-gradient centrifugation with histopaque 1077 (Sigma-Aldrich). For PBMC cultures, RPMI 1640 medium supplemented with 10% fetal calf serum (Invitrogen), 50,000 IU penicillin/L) Invitrogen, and 50 mg streptomycin/L (Invitrogen) was used. Trypan blue was used to assess cell viability. PBMCs were specifically stimulated for 20 h in vitro with different ASFV isolates, adjusted to give 10^6^ of PFU, or RPMI alone and 10 µg/mL of phytohemaglutinin (PHA, Sigma-Aldrich) was used as control of the technique. Any sample scoring ≥ 300 spots/10^6^ PBMCs received a score of 300, which was considered the limit of our assay resolution.

#### 2.4.4. Infection-Inhibition Assay

The infection-inhibition assay was performed as previously described [23] with some modifications. Briefly, 100 µL of each ten-fold dilution of the ASFV strain Ken06.Bus, performed in pig serum from the same healthy donor used to obtain blood-derived monocytes, were mixed with 0.9 mL of heat-inactivated (56 °C, 30 min) autologous serum from donor pig or with immune serum from vaccinated pigs and incubated for 1 h at 37 °C. Blood-derived monocytes cultured in 96-well plates were inoculated with the virus-serum mixtures, and the presence of a cytopathic effect was monitored during 5 days. Finally, cell supernatants were collected on the last day of the experiment to quantify virus loads by qPCR.

### 2.5. Statistical Analysis

Statistical analysis was performed using GraphPad Prism7 software. Differences between groups were determined using unpaired t-test or two-way ANOVA followed by Tukey’s multiple comparison test.

## 3. Results

### Results and Discussion

**Figure 1:**
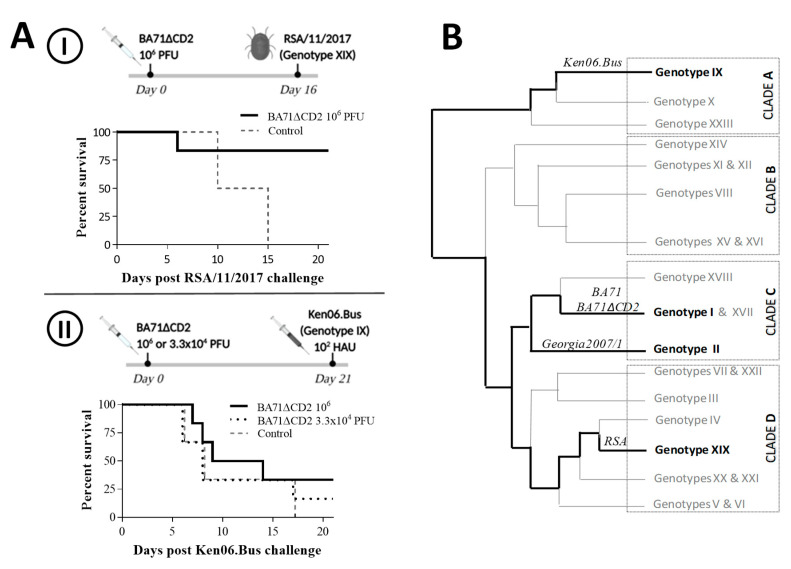
(**A**) Schematic representation of two in vivo experimental designs with the survival rates observed after African swine fever virus (ASFV) challenge and (**B**) ASFV phylogenetic tree showing the genetic distance between different ASFV genotypes and clades described so far, based on Muangkram et al., 2015 [14]. In Italic, strains used in this study: BA71 (genotype I); Georgia2007/1 (genotype II); RSA/11/2017 (genotype XIX); and Ken06.Bus (genotype IX).

Aiming to improve our knowledge about the cross-protective potential of BA71∆CD2, two immunization experiments were performed in parallel (Figure 1).

The first one (Figure 1AI) consisted of a tick-bite challenge experiment using *Ornithodoros* sp soft ticks naturally infected with RSA/11/2017 strain (genotype XIX, clade D). None of the pigs immunized with BA71∆CD2 showed ASF-compatible clinical signs after BA71∆CD2 immunization. After RSA/11/2017 tick-bite challenge, control pigs died of ASF at days 10 and 15 post-challenge (pc), showing clinical signs compatible with acute ASF from day 7 and 14 pc, respectively. Conversely, five out of the six BA71∆CD2-immunized pigs survived the ASFV challenge, with the sixth one succumbing at day 6 pc with evident ASF clinical signs (Figure 1AI). Interestingly, four out of five survivors remained clear of ASF-clinical signs throughout the study, while the fifth survivor showed very mild clinical signs for two days (11–13 pc) and then fully recovered. The presence of ASFV-specific antibodies by ELISA was confirmed in all BA71∆CD2-immunized animals.

Next, this study was extended to Ken06.Bus (Figure 1AII), which is a genotype IX ASFV strain belonging to clade A [17], and the most divergent ASFV strain compared from the BA71∆CD2 vaccine prototype available in our collection (Figure 1B). All the control pigs died between day 6 and 17 pc, while only one (16.6%) and two (33.3%) of the BA71∆CD2-immunized pigs survived, corresponding to the low and high vaccine doses, respectively (Figure 1AII).

**Figure 2:**
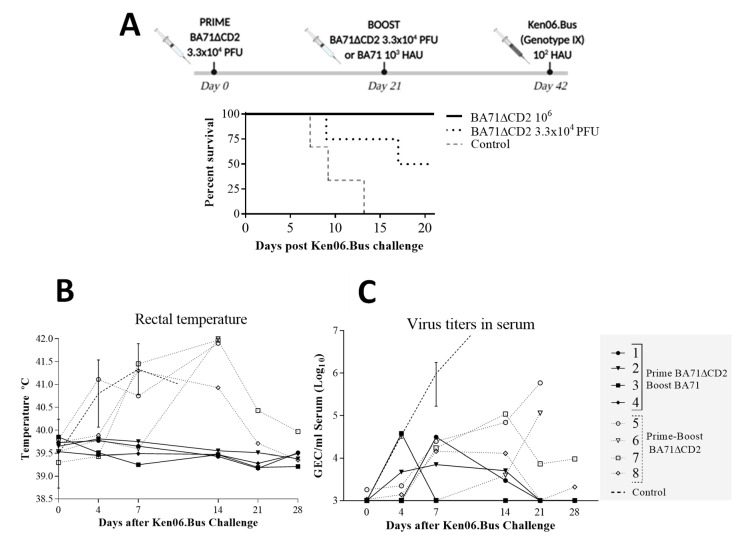
(**A**) Schematic representation the in vivo experimental design with survival rates observed after Ken06.Bus challenge and comparison of the kinetics of (**B**) fever and (**C**) ASFV load in serum, after Ken06.Bus challenge. Data plotted in panels B and C correspond to individual animals inoculated with BA71∆CD2 and boosted with either BA71 (black lines) or BA71∆CD2 (dotted lines). Average and standard deviation values obtained from control animals are also depicted (dashed line). Virus titers are plotted on a logarithmic scale as genome equivalent copies (GEC) per milliliter of serum, being 1 GEC/µL serum the limit of detection of the assay.

We next aimed to improve the protection afforded against Ken06.Bus by using two different prime-boosting strategies (Figure 2A). A strategy previously proved successful for other ASF LAVs [24]. Pigs did not show any clinical sign compatible with ASF and only pig 7, twice immunized with BA71∆CD2, showed low albeit detectable virus in blood (10^3^ GEC/mL of serum) and mild rectal temperature (40.5 °C) at the time of Ken06.Bus (d0 pc in Figure 2B,C, respectively), which was compatible with the residual virulence already described for BA71∆CD2 [16]. Additionally, verifying our previous results, pigs immunized with BA71∆CD2 survived the BA71 challenge [16], remaining free of ASF-compatible clinical signs. Finally, all eleven pigs were IM challenged with a lethal dose of 10^2^ HAU of Ken06.Bus. As expected, control pigs died of acute ASF between days 7 and 14 pc, showing clear clinical signs including fever from day 4 pc (Figure 2B), correlating with the first detection of virus in serum (Figure 2C).

Fifty percent of the pigs immunized twice with BA71∆CD2 survived the Ken06.Bus challenge (Figure 2A) with all of them showing ASF clinical signs, including prolonged fever (Figure 2B). Conversely, 100% of the pigs immunized with BA71∆CD2 and boosted with the virulent BA71 survived the lethal challenge with Ken06.Bus without any apparent clinical signs of the disease with the exception of a very brief peak of low fever in one animal by day 4 pc (Figure 2B).

Despite the different outcome of disease, both immunized groups showed a clear reduction of ASFV in sera of more than three logs in magnitude compared with controls (Figure 2C), confirming that viral load in body fluids does not always correlate with disease outcome [25].

Ideal ASF vaccines should be able to induce both specific antibody and CD8+ T-cell responses, which are essential to obtain solid protection [26,27]. Adaptive immune responses together with the appropriate innate immune triggering [28] explain the solid protection afforded by LAVs, albeit the exact mechanisms exhorted are unknown.

**Figure 3:**
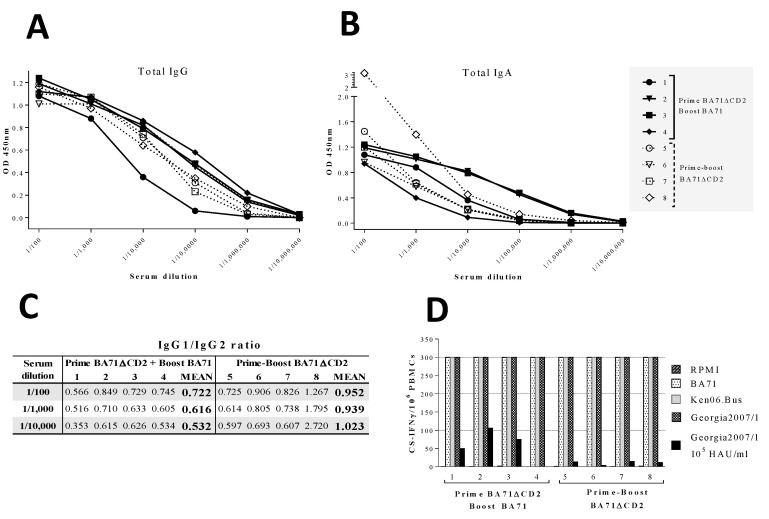
ASFV-specific IgG (**A**) and IgA (**B**) titers found in the blood of pigs at the time of Ken06.Bus challenge, and ASFV-specific IgG1/IgG2 ratios (**C**) and IFNγ-secreting specific T cells found in peripheral blood mononuclear cells (PBMCs) in vitro stimulated with 10^6^ HAU/mL of BA71, Ken06.Bus, or Georgia2007/1. Additionally, PBMCs were stimulated with 10^5^ HAU/mL of Georgia2007/1 (**D**) at this same time point; any sample scoring ≥ 300 spots/10^6^ PBMCs received a score of 300, which was considered the limit of our assay resolution. ASFV-specific antibodies and T-cells were measured by ELISA and ELISPOT (IFN-ϒ secreting cells), respectively.

Despite the differential protection obtained with each one of the immunization regimes, no significant differences were observed in terms of the ASFV-specific IgG titers detected in serum by ELISA at the time of Ken06.Bus challenge, albeit three out of the four pigs boosted with BA71 seemed to show slightly higher levels than that observed in BA71∆CD2-boosted pigs (Figure 3A). No significant differences were observed between groups regarding the ASFV-specific IgA detected in the sera (Figure 3B), but curiously, pig 5 boosted with BA71∆CD2 showed higher OD values than the rest of the animals at high serum concentrations, but the values reached zero at the same dilution at that of the rest of the animals. Limited sample availability impeded confirming this result directly in the mucosal surfaces. Despite not being statistically significant, evident differences were observed between groups when comparing the average ASFV-specific IgG1/IgG2 ratios, independently of the sera dilution used (Figure 3C). Thus, pigs boosted with virulent BA71 showed a clear IgG2 bias, most probably indicating the induction of more efficient Th1-like responses [29] compared with BA71∆CD2-boosted animals, which showed an equilibrated IgG1/IgG2 ratio (almost 1 in average).

Despite sera from immunized pigs partially inhibiting ASFV infection in vitro, the inhibition observed was indistinguishably between both immunization groups, and most probably too weak (not shown) to explain the cross-protection observed [13]. The lack of CD2v (the ASFV hemagglutinin) in BA71∆CD2 might explain this result, since a tight correlation was observed between in vitro infection inhibition and the ability of sera to inhibit the ASFV-specific hemagglutination [30], which is an activity not found in any of the pig groups at any time tested (not shown). Recent studies have confirmed CD2v and the lectin-like gene of ASFV as crucial antigens defining ASFV serotypes and cross-protection [31]. Alternative immunological mechanisms and ASFV determinants might account for the protection afforded by natural non-hemadsorbing LAVs [15,32,33,34,35] and for the cross-protection observed after experimental immunization with BA71∆CD2 in the absence of CD2v [16]. Comparative studies could be performed in the future to identify qualitative differences in the antibody responses induced, including neutralization, complement lysis, and antibody-dependent cellular cytotoxicity (ADCC) assays, which were previously described in pigs surviving the ASFV infection [36,37].

Finally, all pigs, independently of the immunization group, showed a large number of virus-specific T-cells in PBMCs quantified by IFNγ-ELISPOT after stimulation with 10^6^ HAU/mL of BA71, Ken06.Bus or Georgia2007/1, all of them out of the limit of visual resolution. However, the stimulation of PBMCs with 10 times less Georgia2007/1 (10^5^ HAU/mL) exclusively induced significant IFNγ-ELISPOT responses in PBMCs from three out of the four pigs inoculated with BA71∆CD2 plus BA71 (Figure 3D).

Far from conclusive, this result seems to confirm the Th1 bias showed by the ELISA results and might reflect quantitative and/or qualitative differences in the T-cell responses induced, as described before to explain the cross-protection afforded by experimental immunization of pigs with the non-virulent OURT88/3 followed by the closely related virulent OURT88/1 [21]. Future work should concentrate its attention on deeply characterizing the T-cell responses induced by the different immunization regimes, which is a crucial arm of the immune response involved in both protection and cross-protection against ASFV strains [16,26,28].

We believe that the superior protection afforded by the BA71-boosting is most probably multifactorial, contributing innate immunity and both B and T cell responses, as it has been described in different mouse models [38,39].

The ability of the virulent BA71 ASFV isolate to systemically travel attached to red blood cells much more efficiently than BA71∆CD2 (without CD2v) might provide advantages at the time of infecting distant antigen-presenting cells (APCs), thus amplifying the protective signature primed by BA71∆CD2 and perhaps even unmasking subdominant protective epitopes [40]. Although the specific T-cell responses detected do not seem very dramatic, we should take into account that these differences are observed despite boosting pigs with 10 times less BA71 than BA71∆CD2. Taking advantage of the cross-protection afforded by BA71∆CD2 [16], we will try to reproduce the protection afforded against Ken06.Bus by previous immunization with BA71∆CD2 plus Georgia2007/1. If as expected, pigs surviving Georgia2007/1 heterologous challenge behave similarly to those surviving BA71 (homologous strain), this in vivo model could be used to experimentally mimic very complex epidemiological realities, such as those found in some regions of Eastern Africa, where many different strains can concomitantly circulate [41]. Lessons learned from the Spanish epidemic period [5,13] recommend being prepared for the potential appearance of new ASFV variants in the rest of the world, which might need vaccines à la carte or cross-protective vaccine prototypes.
